# Supplementary material for: Visual–Tactile Perception of Biobased Composites
Source: Materials (Basel). 2023 Feb 23;16(5):1844. doi: 10.3390/ma16051844 (PMC10004420; doi:10.3390/ma16051844)
Supplement: Supplementary file 1 [file materials-16-01844-s001.zip › materials-2212938-supplementary.pdf]

Supplementary Data

Materials used in the study

No.	Sample Name	Matrix	Reinforcement
1	Cellulose + Wood	Microcrystalline Cellulose (MCC) (Sigma Aldrich)	40 mass% wood fibre (*Pinus radiata*), 1.64 ± 0.7 mm average length.
2	Rayon	Thermoplastic Starch (TPS) (Shandong Zoneyoung Chemicals)	20 mass% Rayon™ 700 rayon fibre, 30 mm average length
3	Leather	Leather	
4	NW Coir	*WEST SYSTEM 105* epoxy resin	15 mass% non-woven coir fleece, 30-50mm fibre length
5	NW Sisal	*WEST SYSTEM 105* epoxy resin	15 mass% non-woven sisal fleece
6	Poplar	Poplar Wood	
7	TW Cotton	*WEST SYSTEM 105* epoxy resin	25 mass% twill-weave cotton
8	TW Flax1	*WEST SYSTEM 105* epoxy resin	25 mass% twill-weave flax
9	TW Flax2	Thermoplastic Starch (TPS) (Shandong Zoneyoung Chemicals)	25 mass% twill-weave flax
10	UD Flax	Thermoplastic Starch (TPS) (Shandong Zoneyoung Chemicals)	25 mass% unidirectional flax
11	Walnut	Walnut Wood	

Cellulose+ Wood composite was prepared by mixing the wood fibre with Microcrystalline dissolved in an ionic liquid as per the methodology outlined by Huber (2012).

For TPS based composites, starch powder was mixed with water at 1:6 ratio and heated to 90^{0}C to form thermoplastic starch gel. Fibre reinforcement was mixed with this gel and then clamped between MDF plates to form samples.

For epoxy-based composites, the fibres were mixed with the resin and hardener, and then pressed between two stainless steel plates at 0.2 MPa.

Table S1: *Correlated attribute pairs with statistical significance (95% confidence level)*

Positive Correlations			Negative Correlations		
Attribute Pair		Rho	Attribute Pair		Rho
Ordinary	Simple	0.982	Ordinary	Complex	-0.982
Unusual	Complex	0.980	Simple	Unusual	-0.982
Valuable	Beautiful	0.918	Ugly	Valuable	-0.882
Ugly	Artificial	0.836	Worthless	Beautiful	-0.858
Worthless	Ugly	0.817	Ugly	Natural	-0.845
Beautiful	Natural	0.791	Artificial	Beautiful	-0.822
Boring	Simple	0.752	Artificial	Valuable	-0.776
Valuable	Natural	0.736	Boring	Complex	-0.743

Interesting	Complex	0.719	Simple	Interesting	-0.733
Worthless	Artificial	0.709	Cold	Natural	-0.702
Ordinary	Boring	0.685	Boring	Unusual	-0.691
Unusual	Interesting	0.680	Rough	Valuable	-0.682
Smooth	Strong	0.667	Ordinary	Interesting	-0.674
Smooth	Valuable	0.665	Smooth	Weak	-0.656
Weak	Rough	0.645	Worthless	Natural	-0.648
Weak	Hot	0.644	Smooth	Complex	-0.630
Smooth	Simple	0.639	Rough	Strong	-0.624
Cold	Artificial	0.629	Cold	Weak	-0.624
Boring	New	0.606	New	Interesting	-0.620
			Simple	Rough	-0.606

Note: $p<0.05$, $df=18$

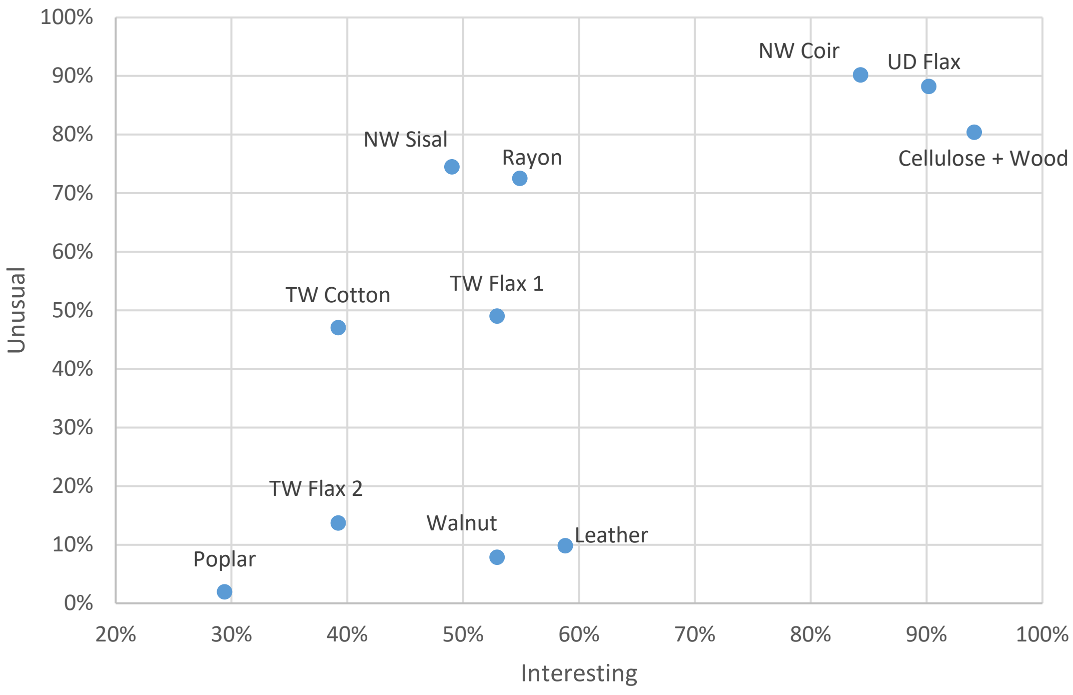

Figure S1: *Material perception for Unusual-Interesting (visual + tactile perception)*

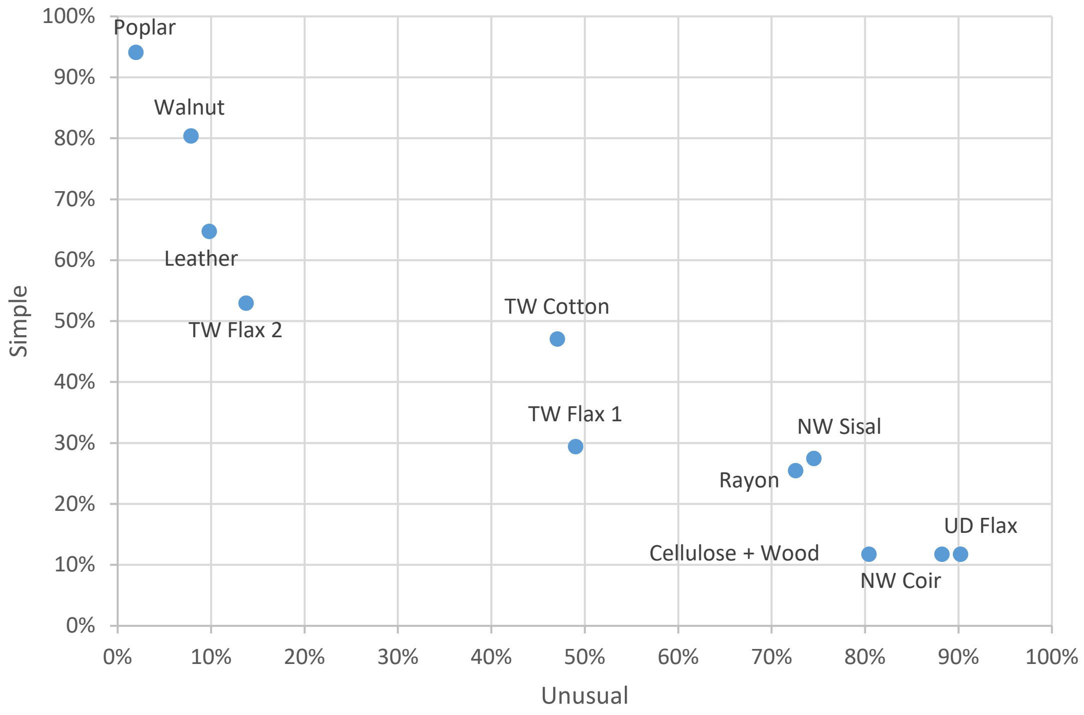

***Figure S2**: Material perception for Unusual-Simple (visual + tactile perception)*

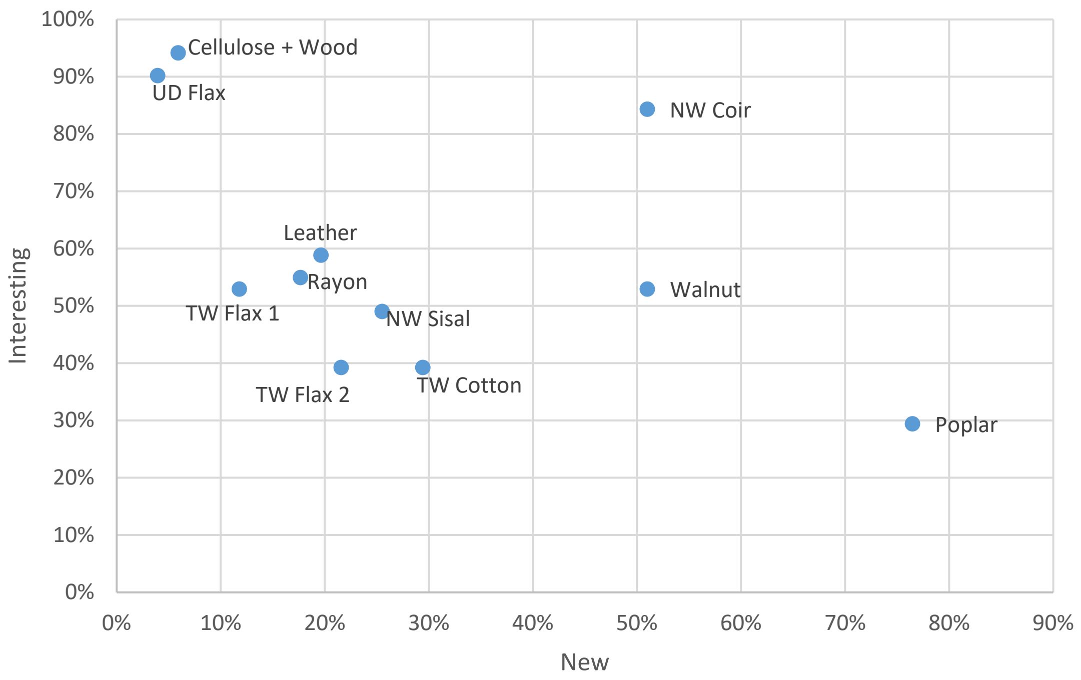

***Figure S3**: Material perception for New-Interesting (visual + tactile perception)*

***Table S2**: Inferences from Combining Attribute Correlations*

Positive Correlations			Negative Correlations			Inferences	
Ordinary	Simple	0.982	Ordinary	Complex	-0.982	Complexity ~ Unusual	Complexity ~ Novelty ~ Interesting
Unusual	Complex	0.980	Simple	Unusual	-0.982		
Ordinary	Boring	0.685	Boring	Unusual	-0.691	Interesting ~ Unusual	
Unusual	Interesting	0.68	Ordinary	Interesting	-0.674		
Boring	Simple	0.752	Boring	Complex	-0.743	Complexity ~ Interesting	
Interesting	Complex	0.719	Simple	Interesting	-0.733		
Valuable	Beautiful	0.918	Ugly	Valuable	-0.882	Beautiful ~ Valuable	Beauty ~ Worth ~ Natural
Worthless	Ugly	0.817	Worthless	Beautiful	-0.858		
Valuable	Natural	0.736	Artificial	Valuable	-0.776	Natural ~ Valuable	
Worthless	Artificial	0.709	Worthless	Natural	-0.648		
Ugly	Artificial	0.836	Ugly	Natural	-0.845	Natural ~ Beautiful	
Beautiful	Natural	0.791	Artificial	Beautiful	-0.822		
Cold	Artificial	0.629	Cold	Natural	-0.702	Artificial ~ Cold	Artificial ~ Cold ~ Strong
Weak	Hot	0.644	Cold	Weak	-0.624	Hot ~ Weak	
Boring	New	0.606	New	Interesting	-0.62	New ~ Boring	New ~ Boring
Smooth	Valuable	0.665	Rough	Valuable	-0.682	Smooth ~ Valuable	Smooth ~ Worth ~ Simple ~ Strong
Smooth	Strong	0.667	Smooth	Weak	-0.656	Smooth ~ Strong	
Weak	Rough	0.645	Rough	Strong	-0.624		
Smooth	Simple	0.639	Smooth	Complex	-0.63	Smooth~ Simple	
			Simple	Rough	-0.606		

***Table S3**: Materials and corresponding attribute ratings (bimodal perception)*

	Aged-New	Complex-Simple	Interesting-Boring	Natural-Artificial	Unusual-Ordinary	Beautiful-Ugly	Valuable-Worthless	Strong-Weak	Rough-Smooth	Hot-Cold
**Cellulose + Wood**	Aged	Complex	Interesting	Natural	Unusual	Beautiful	Can't Say	Can't Say	Rough	Can't Say
**Rayon**	Aged	Complex	Interesting	Natural	Unusual	Ugly	Worthless	Weak	Rough	Hot
**Leather**	Aged	Simple	Interesting	Natural	Ordinary	Beautiful	Valuable	Strong	Smooth	Hot
**NW Coir**	New	Complex	Interesting	Natural	Unusual	Beautiful	Can't Say	Strong	Smooth	Can't Say
**NW Sisal**	Can't Say	Complex	Can't Say	Can't Say	Unusual	Ugly	Worthless	Strong	Smooth	Can't Say
**Poplar**	New	Simple	Boring	Natural	Ordinary	Beautiful	Worthless	Can't Say	Smooth	Can't Say
**TW Cotton**	Can't Say	Can't Say	Boring	Artificial	Can't Say	Ugly	Worthless	Strong	Smooth	Cold
**TW Flax 1**	Aged	Complex	Interesting	Artificial	Can't Say	Ugly	Worthless	Strong	Smooth	Can't Say
**TW Flax 2**	Aged	Simple	Can't Say	Can't Say	Ordinary	Can't Say	Worthless	Can't Say	Rough	Hot
**UD Flax**	Aged	Complex	Interesting	Natural	Unusual	Beautiful	Can't Say	Can't Say	Rough	Hot
**Walnut**	New	Simple	Interesting	Natural	Ordinary	Beautiful	Valuable	Strong	Smooth	Can't Say

Note: cells in yellow - Looks Like; cells in green – Definitely

***Table S4**: Ranking of material samples against each attribute (bimodal perception)*

	Aged	Complex	Interesting	Natural	Unusual	Beautiful	Valuable	Strong	Rough	Hot	New	Simple	Boring	Artificial	Ordinary	Ugly	Worthless	Weak	Smooth	Cold
**Cellulose + Wood**	2	2	1	5	3	5	6	7	4	6	10	9	11	6	9	8	7	4	8	6
**Rayon**	6	4	5	6	5	9	9	11	3	1	8	8	8	5	7	4	2	1	9	9
**Leather**	4	9	4	4	9	3	2	4	11	4	7	3	7	7	3	9	11	6	1	9
**NW Coir**	10	1	3	7	1	4	4	3	7	7	2	9	9	7	11	7	9	10	6	4
**NW Sisal**	9	5	8	9	4	11	11	6	5	8	5	7	4	3	8	1	1	7	7	3
**Poplar**	11	11	11	3	11	2	3	8	10	8	1	1	1	9	1	10	6	5	2	5
**TW Cotton**	7	7	9	11	7	10	8	4	8	11	4	5	2	1	5	2	3	9	4	1
**TW Flax 1**	2	6	6	10	6	8	7	2	6	10	9	6	5	2	5	3	5	8	5	2
**TW Flax 2**	5	7	9	8	8	7	10	9	1	1	6	4	3	4	4	5	3	2	11	8
**UD Flax**	1	3	2	2	2	6	5	10	2	1	11	9	10	10	9	6	7	3	10	11
**Walnut**	8	10	6	1	10	1	1	1	9	5	2	2	6	10	2	11	10	11	2	7

Note: Top rankings are highlighted in green and lowest ranks are highlighted in red

***Table S5**: Fraction of Positive Responses against Each Attribute*

	**Aged**	**Complex**	**Interesting**	**Natural**	**Unusual**	**Beautiful**	**Valuable**	**Strong**	**Rough**	**Hot**	**New**	**Simple**	**Boring**	**Artificial**	**Ordinary**	**Ugly**	**Worthless**	**Weak**	**Smooth**	**Cold**
**Cellulose + Wood**	74.5%	80.4%	94.1%	72.5%	80.4%	56.9%	25.5%	49.0%	66.7%	33.3%	5.9%	11.8%	2.0%	23.5%	11.8%	23.5%	43.1%	39.2%	21.6%	31.4%
**Rayon**	52.9%	64.7%	54.9%	68.6%	72.5%	23.5%	15.7%	9.8%	84.3%	58.8%	17.6%	25.5%	31.4%	25.5%	17.6%	54.9%	64.7%	74.5%	13.7%	15.7%
**Leather**	66.7%	19.6%	58.8%	74.5%	9.8%	70.6%	72.5%	56.9%	2.0%	54.9%	19.6%	64.7%	33.3%	19.6%	80.4%	15.7%	7.8%	33.3%	96.1%	15.7%
**NW Coir**	23.5%	84.3%	84.3%	66.7%	90.2%	58.8%	33.3%	62.7%	23.5%	27.5%	51.0%	11.8%	9.8%	19.6%	7.8%	29.4%	35.3%	19.6%	72.5%	41.2%
**NW Sisal**	39.2%	56.9%	49.0%	41.2%	74.5%	11.8%	5.9%	51.0%	27.5%	25.5%	25.5%	27.5%	43.1%	37.3%	13.7%	76.5%	70.6%	31.4%	70.6%	45.1%
**Poplar**	2.0%	3.9%	29.4%	82.4%	2.0%	76.5%	35.3%	47.1%	3.9%	25.5%	76.5%	94.1%	62.7%	9.8%	92.2%	9.8%	52.9%	35.3%	90.2%	39.2%
**TW Cotton**	49.0%	35.3%	39.2%	17.6%	47.1%	15.7%	17.6%	56.9%	19.6%	9.8%	29.4%	47.1%	52.9%	68.6%	41.2%	66.7%	62.7%	21.6%	80.4%	66.7%
**TW Flax 1**	74.5%	52.9%	52.9%	19.6%	49.0%	27.5%	21.6%	66.7%	25.5%	23.5%	11.8%	29.4%	41.2%	64.7%	41.2%	62.7%	56.9%	25.5%	74.5%	49.0%
**TW Flax 2**	56.9%	35.3%	39.2%	49.0%	13.7%	47.1%	13.7%	43.1%	100.0%	58.8%	21.6%	52.9%	49.0%	35.3%	76.5%	39.2%	62.7%	45.1%	0.0%	25.5%
**UD Flax**	88.2%	78.4%	90.2%	84.3%	88.2%	54.9%	31.4%	41.2%	92.2%	58.8%	3.9%	11.8%	5.9%	7.8%	11.8%	37.3%	43.1%	41.2%	7.8%	5.9%
**Walnut**	41.2%	15.7%	52.9%	88.2%	7.8%	98.0%	74.5%	86.3%	5.9%	45.1%	51.0%	80.4%	37.3%	7.8%	84.3%	2.0%	15.7%	5.9%	90.2%	29.4%

Table S6: *Spearman's Rank Correlation Coefficient for Various Attribute Pairs*

	Aged	Complex	Interesting	Natural	Unusual	Beautiful	Valuable	Strong	Rough	Hot	New	Simple	Boring	Artificial	Ordinary	Ugly	Worthless	Weak	Smooth	Cold
**Aged**																				
**Complex**	0.301																			
**Interesting**	0.547	.719*																		
**Natural**	0.041	-0.205	0.338																	
**Unusual**	0.278	.980**	.680*	-0.218																
**Beautiful**	-0.159	-0.351	0.183	.791**	-0.391															
**Valuable**	-0.096	-0.328	0.265	.736**	-0.327	.918**														
**Strong**	-0.210	-0.212	-0.030	-0.141	-0.173	0.278	0.428													
**Rough**	0.465	0.588	0.228	-0.191	0.555	-0.518	-.682*	-.624*												
**Hot**	0.378	0.152	0.402	0.543	0.087	0.216	0.055	-0.567	0.492											
**New**	-.936**	-0.507	-.620*	0.018	-0.478	0.333	0.278	0.379	-0.601	-0.396										
**Simple**	-0.377	-.989**	-.733*	0.174	-.982**	0.385	0.330	0.257	-.606*	-0.158	0.579									
**Boring**	-0.510	-.743**	-.986**	-0.373	-.691*	-0.173	-0.218	0.105	-0.318	-0.483	.606*	.752**								
**Artificial**	0.059	0.101	-0.365	-.959**	0.091	-.822**	-.776**	0.066	0.205	-0.490	-0.119	-0.088	0.393							
**Ordinary**	-0.252	-.982**	-.674*	0.237	-.995**	0.397	0.338	0.172	-0.539	-0.065	0.453	.982**	.685*	-0.117						
**Ugly**	0.123	0.342	-0.215	-.845**	0.400	-.982**	-.882**	-0.159	0.464	-0.299	-0.273	-0.367	0.218	.836**	-0.397					
**Worthless**	-0.130	0.101	-0.500	-.648*	0.110	-.858**	-.918**	-0.446	0.507	-0.185	-0.064	-0.115	0.438	.709*	-0.117	.817**				
**Weak**	0.419	0.178	0.110	0.127	0.100	-0.227	-0.409	-.934**	.645*	.644*	-0.551	-0.211	-0.173	-0.009	-0.091	0.109	0.365			
**Smooth**	-0.384	-.630*	-0.240	0.173	-0.597	0.487	.665*	.667*	-.989**	-0.505	0.537	.639*	0.333	-0.162	0.586	-0.433	-0.490	-.656*		
**Cold**	-0.411	-0.037	-0.435	-.702*	0.009	-0.346	-0.251	0.555	-0.301	-.949**	0.411	0.069	0.487	.629*	-0.037	0.433	0.325	-.624*	0.311	

** Correlation is significant at the 0.01 level (2-tailed).
* Correlation is significant at the 0.05 level (2-tailed).

Note: ■ *Significant (Inverse) correlations - correlations between bipolar adjectives on the same attribute scale,* ■ *Significant (Negative) correlations and* ■ *Significant (Positive) correlations.*

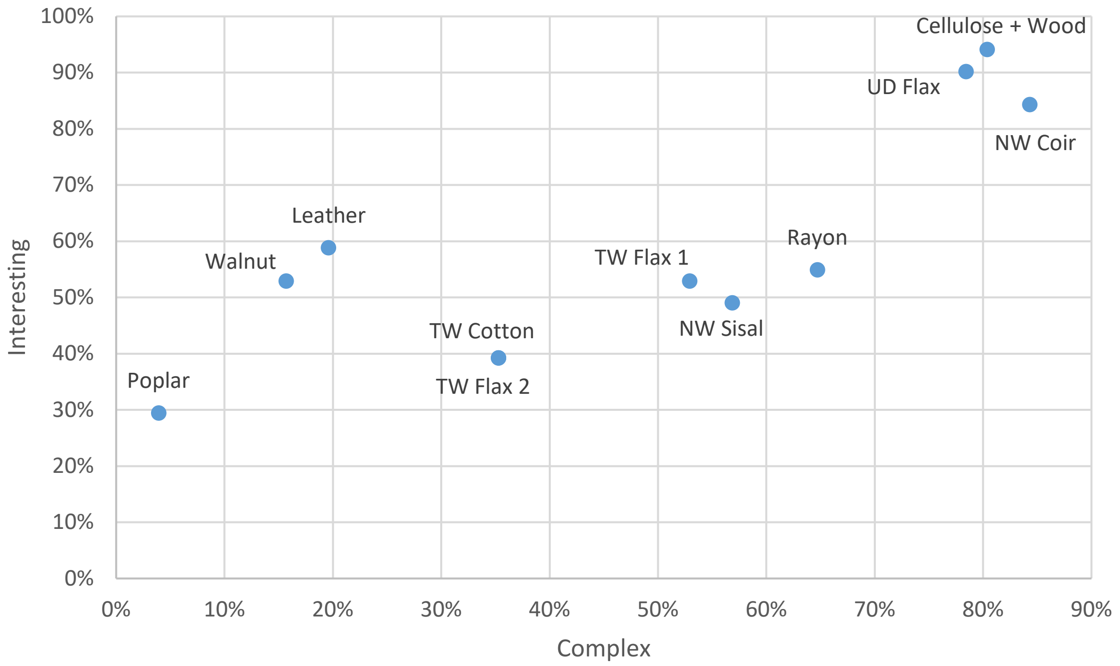

Figure S4: *Material perception for Complex-Interesting (visual + tactile perception)*

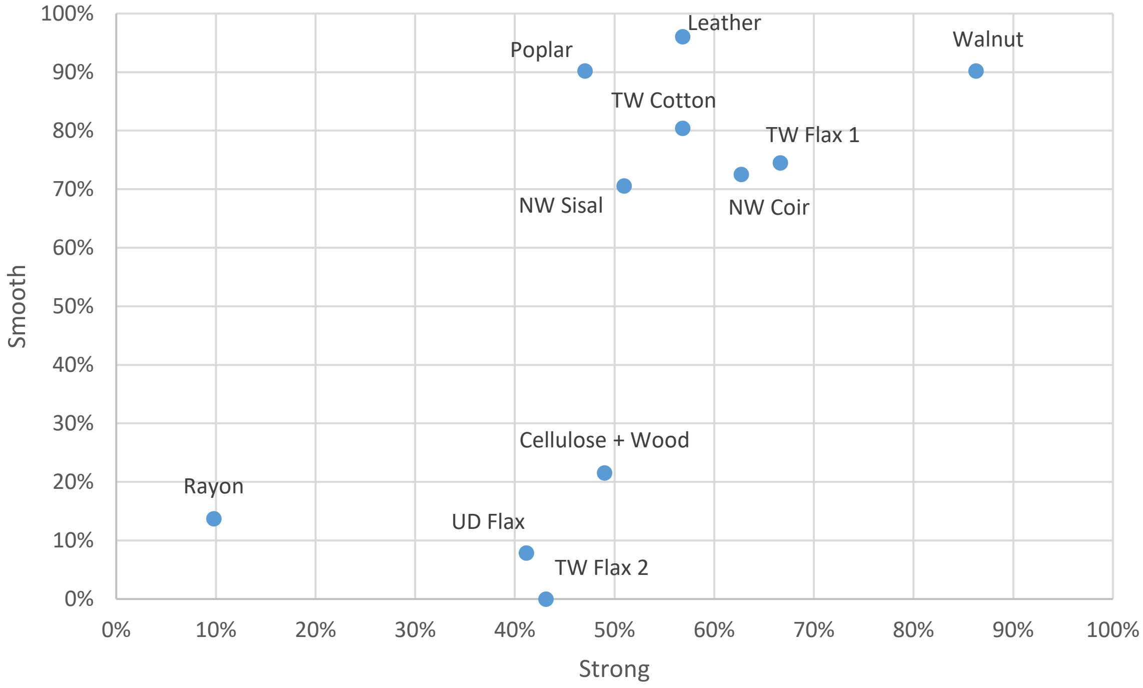

Figure S5: *Material perception for Smooth-Strong (visual + tactile perception)*

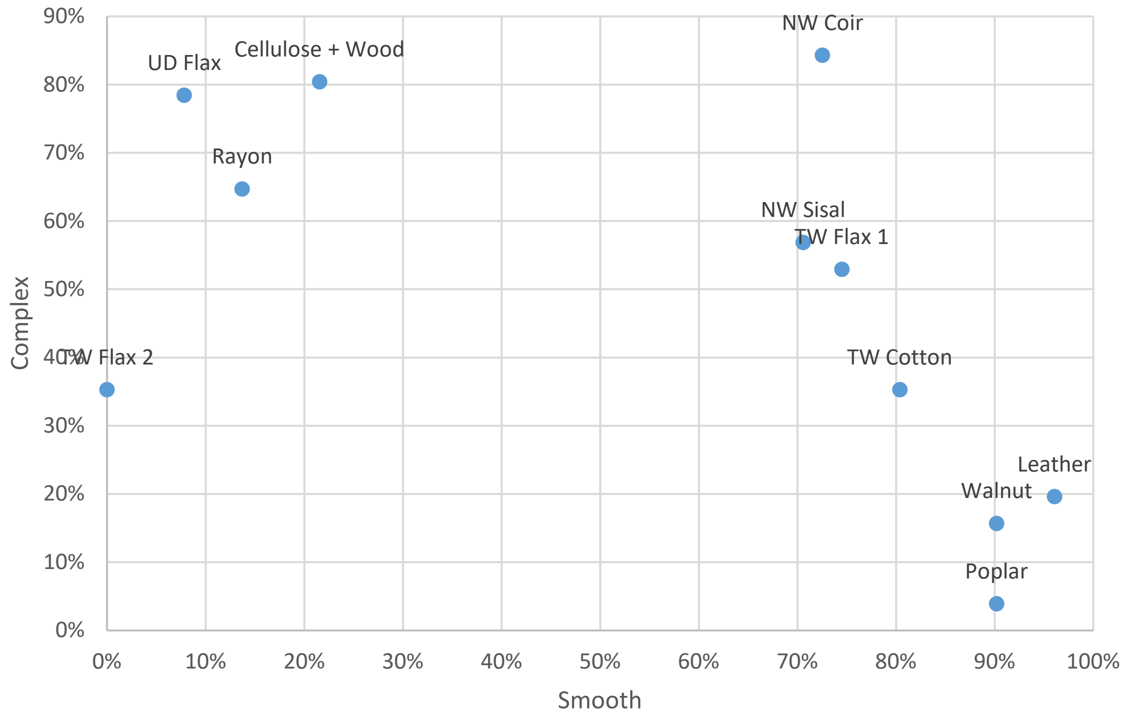

***Figure S6**: Material perception for Smooth-Valuable (visual + tactile perception)*

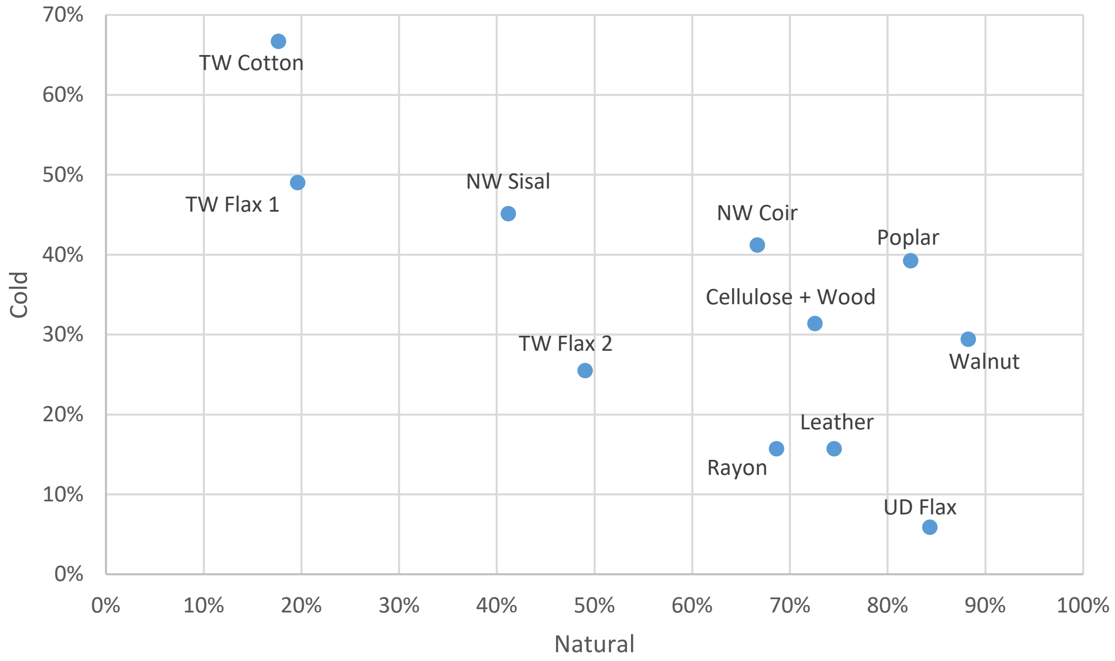

***Figure S7**: Material perception for Cold-Natural (visual + tactile perception)*

***Table S7**: Uncertainty in Perception for Materials and Attribute Pairs*

Material Sample	Uncertainty (%)	Attribute Pair	Uncertainty (%)
NW Sisal	17.7%	Hot-Cold	31.4%
TW Cotton	15.5%	Valuable-Worthless	23.7%
Cellulose + Wood	15.3%	Aged-New	21.8%
Rayon	15.3%	Strong-Weak	15.7%
NW Coir	14.7%	Beautiful-Ugly	14.1%
TW Flax 2	13.5%	Complex-Simple	11.6%
Poplar	12.9%	Natural-Artificial	11.6%
TW Flax 1	12.9%	Interesting-Boring	8.6%
Leather	12.8%	Unusual-Ordinary	8.6%
UD Flax	11.6%	Rough-Smooth	3.1%
Walnut	8.0%		

***Table S8:** Attribute pairs and number of statistically significant correlations*

**Attributes** *(with the number of significant correlations)*	
Hot (1)	Cold (3)
Valuable (6)	Worthless (4)
Aged (0)	New (2)
Strong (2)	Weak (4)
Beautiful (4)	Ugly (4)
Complex (5)	Simple (6)
Natural (5)	Artificial (5)
Interesting (5)	Boring (5)
Unusual (4)	Ordinary (4)
Rough (4)	Smooth (5)
